# Non-invasive *in vivo* imaging of arthritis in a collagen-induced murine model with phosphatidylserine-binding near-infrared (NIR) dye

**DOI:** 10.1186/s13075-015-0565-x

**Published:** 2015-03-09

**Authors:** Marion M Chan, Brian D Gray, Koon Y Pak, Dunne Fong

**Affiliations:** Department of Microbiology and Immunology, Temple University School of Medicine, 3400 North Broad Street, Philadelphia, PA 19140 USA; Molecular Targeting Technologies, Inc., 833 Lincoln Avenue, West Chester, PA 19380 USA; Department of Cell Biology and Neuroscience, Rutgers, The State University of New Jersey, 604 Allison Road, Piscataway, NJ 08854 USA

## Abstract

**Introduction:**

Development of non-invasive molecular imaging techniques that are based on cellular changes in inflammation has been of active interest for arthritis diagnosis. This technology will allow real-time detection of tissue damage and facilitate earlier treatment of the disease, thus representing an improvement over X-rays, which detect bone damage at the advanced stage. Tracing apoptosis, an event occurring in inflammation, has been a strategy used. PSVue 794 is a low-molecular-weight, near-infrared (NIR)-emitting complex of bis(zinc^2+^-dipicolylamine) (Zn-DPA) that binds to phosphatidylserine (PS), a plasma membrane anionic phospholipid that becomes flipped externally upon cell death by apoptosis. In this study, we evaluated the capacity of PSVue 794 to act as an *in vivo* probe for non-invasive molecular imaging assessment of rheumatoid arthritis (RA) via metabolic function in murine collagen-induced arthritis, a widely adopted animal model for RA.

**Methods:**

Male DBA/1 strain mice were treated twice with chicken collagen type II in Freund’s adjuvant. Their arthritis development was determined by measuring footpad thickness and confirmed with X-ray analysis and histology. *In vivo* imaging was performed with the NIR dye and the LI-COR Odyssey Image System. The level of emission was compared among mice with different disease severity, non-arthritic mice and arthritic mice injected with a control dye without the Zn-DPA targeting moiety.

**Results:**

Fluorescent emission correlated reliably with the degree of footpad swelling and the manifestation of arthritis. *Ex vivo* examination showed emission was from the joint. Specificity of binding was confirmed by the lack of emission when arthritic mice were given the control dye. Furthermore, the PS-binding protein annexin V displaced the NIR dye from binding, and the difference in emission was numerically measurable on a scale.

**Conclusions:**

This report introduces an economical alternative method for assessing arthritis non-invasively in murine models. Inflammation in feet and ankles can be measured longitudinally using the PSVue 794 probe for cell death and with a commonly available multipurpose imager. This technique provides metabolic and functional information that anatomical measurement of footpad swelling or visual determination of arthritic index cannot. It also may decrease the number of animals required per experiment because tissue damage will not necessarily require evaluation by harvesting joints for histology.

## Introduction

According to the American College of Rheumatology, the autoimmune disease rheumatoid arthritis (RA) affects 1.3 million adults in the United States [1]. Currently, standard diagnosis relies on clinical symptoms such as tenderness, stiffness, swelling and pain. More objective measurement has to wait for the disease to advance to bone damage, which can be imaged by radiography [2]. As disease-modifying drugs are most effective when used early in the disease process, there is a need to develop new techniques suitable for early imaging of arthritis in real time. Magnetic resonance imaging detects earlier stages of arthritis. However, it faces a challenge in diagnosing synovitis (that is, inflammation of the membrane) in the small-sized phalangeal joints [3]. In humans, a movement to develop molecular imaging by metabolic changes has begun following positive outcomes in animal models. Targets and probes include protease activity, endothelial activation, macrophage accumulation in the inflamed joints and cell death [4-8]. The current status of molecular imaging of RA has been reviewed comprehensively by Put *et al.* [9].

For RA, murine collagen-induced arthritis (CIA) is a preclinical small-animal model that has been used routinely for designing and testing new therapies [10-15]. In this model, arthritis is induced by immunization with heterologous collagen to generate antigen–antibody complexes that deposit on the joints. The chronic inflammatory state presents as synovitis with features including cytokine production, endothelial activation, neutrophil and macrophage infiltration, fibroblast hyperplasia and cartilage and bone damage [14,16]. A counterbalancing proresolution mechanism underlies the pathogenesis whereby apoptosis of the immune activated cells leads to remission and termination of the inflammatory response [17]. The pathogenic mechanisms and cellular dynamics during disease onset and progression in mice have been characterized extensively and are regarded as similar to humans [18]. These developments have increased our knowledge of the immune reactions and metabolic pathways involved in autoimmunity-induced injuries. Wide acceptance of the CIA model is illustrated by the fact that JAX-West (Sacramento, CA, USA), Charles River Laboratories (Wilmington, MA, USA), MD Biosciences (St Paul, MN, USA), Hooke Laboratories (Lawrence, MA, USA) and Washington Biotechnology (Seattle, WA, USA) all offer efficacy testing of commercial compounds for arthritis based on this model. The standard readouts for disease activity are anatomical: ankle and paw swelling over time, clinical signs of inflammation (redness and stiffness) and histological scoring of joint damage [11-13].

Cell proliferation and death are both intricate and highly regulated steps in an inflammatory reaction [19]. At the height of inflammation, tissue damage leads to cell death. The immunoreactive cells are cleared by apoptosis as well [20,21]. For example, neutrophils are a class of short-lived immunoreactive cells that infiltrate inflamed sites in large numbers and die by apoptosis during its resolution [22]. Detection of apoptosis is one of the novel targets for visualizing functional changes at the cellular level [2].

Phosphatidylserine (PS) is an anionic, negatively charged phospholipid located internally within the cytoplasmic leaflet of the plasma membrane. It is a molecular marker of cell death because it is externalized during apoptosis [23,24]. The molecule then becomes a recognition signal for phagocytic binding and removal of the expended cell. Annexin V is a small protein (36 kDa in size) that binds to PS and has been used as a standard marker for apoptosis *in vitro* and in flow cytometry [25]. However, it has limitations *in vivo*, where the microenvironment is more variable and complex [26,27]. The search for alternate markers suitable for *in vivo* imaging of cell death continues.

**Figure 1:**
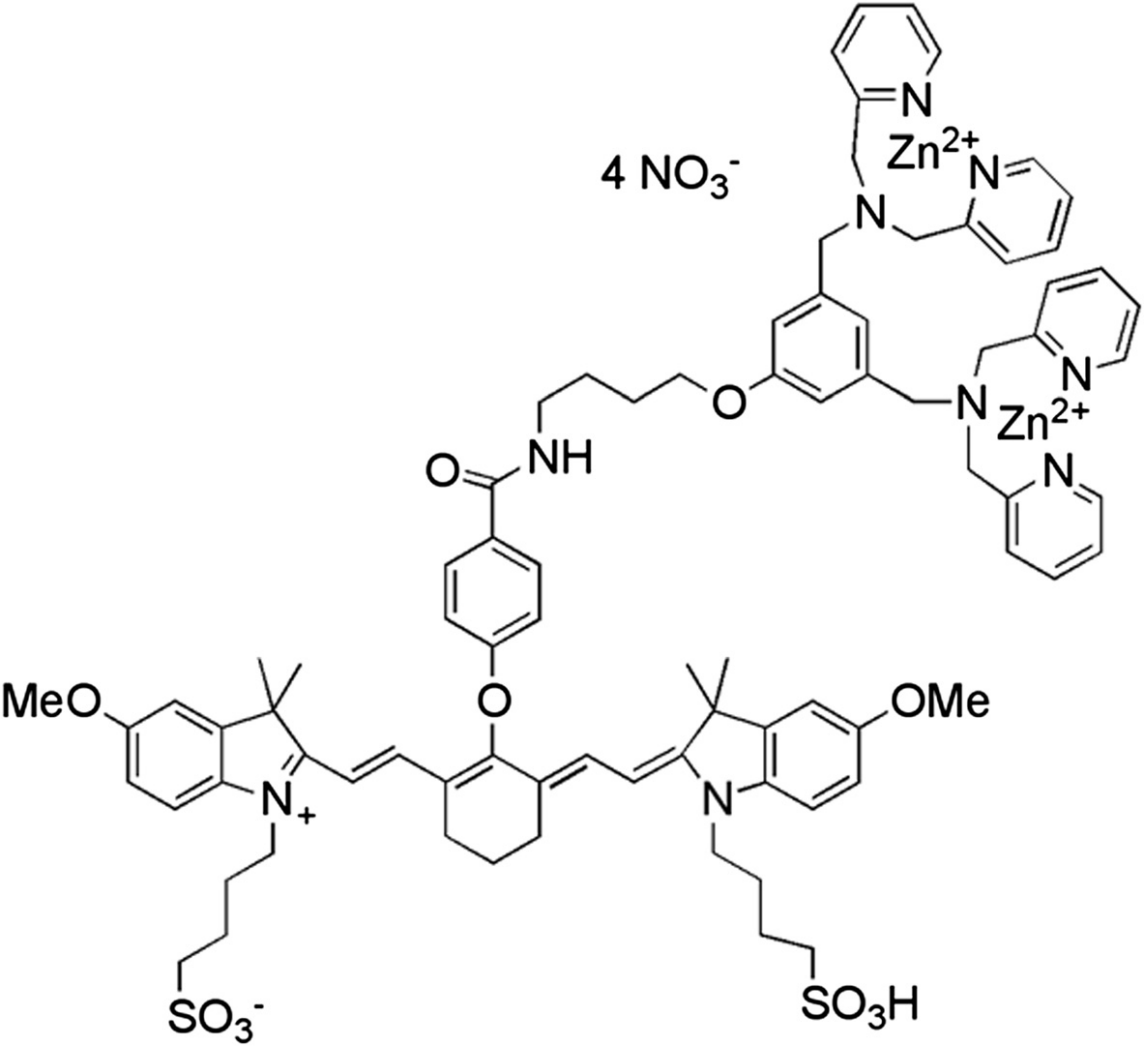
**Chemical structure of PSVue 794.**

Near-infrared (NIR) dyes are promising modalities for non-invasive molecular imaging. PSVue®794 (hereafter referred as PSVue®794; USA) is a low-molecular-weight compound (1,837.6 g/mol) comprising a bis(zinc^2+^-dipicolylamine) (Zn-DPA) complex attached to a fluorescent dye with an excitation maximum of 794 nm and an emission maximum of 810 nm (Figure 1). Its photophysical properties, spectra and quantum yield, absorption and emission curves, are available in the supplementary material in the publication by Leevy *et al.* [28]. PSVue 794 binds to anionic phospholipids, including PS, by the Zn-DPA targeting moiety and thus can be used as a marker of cell death, both apoptotic and necrotic [29]. In fact, the zinc coordination complex has been documented to distinguish dead and dying mammalian cells from live ones *in vitro* and *in vivo. In vitro*, it co-localizes with annexin V in Jurkat cells that are induced for apoptosis, but not in live cells [29,30]. It also co-localizes with propidium iodide in murine hippocampal HT22 cells induced for cell death, but not in healthy cells [31]. *In vivo*, its binding to anionic phospholipids on apoptotic cells has been demonstrated with tumors killed with the tyrosine kinase inhibitor sunitinib [32] and in thymi from dexamethasone-treated rats [33]. Interestingly, PSVue 794 also binds to bacteria with anionic phospholipids, such as *Staphylococcus aureus* and *Escherichia coli* [28 and supplementary material therein].

PSVue 794 exhibits fluorescence at the NIR region, where tissue absorption and non-specific backgrounds are low [28]. For *in vivo* imaging, biodistribution studies in rodent models have shown that intravenously administered molecules accumulate in the liver and kidney when unbound and are cleared from the system within 48 hours [30,33]. Intravenously injected dye labels epidermal carcinoma xenografted into nude mice treated with the drug sunitinib, but not the untreated counterpart. Its selectivity has been established with a non-specific control probe that contains the same fluorophore, but without the Zn-DPA targeting moiety [32]. Zn-DPA, with various labels, has been used for *in vivo* imaging of apoptotic and necrotic regions in liver, thymus, mammary and prostate tumors, cerebral artery occlusion and acute cell death conditions in rodents [29-34].

In the present study, we evaluated whether the cell death targeting properties of PSVue 794 could be used to non-invasively visualize the degree of autoimmune-mediated inflammation in the murine CIA model *in vivo*. The method described here correlates with anatomical changes and allows semiquantitative comparison using an economical fluorescence imager that is commonly available in most laboratories.

## Methods

### Induction of arthritis

The experimental protocol used in this study was approved by the Temple University Institutional Animal Care and Use Committee. RA was induced in 6- to 8-week-old male DBA/1 mice (The Jackson Laboratory, Bar Harbor, ME, USA) with 50 μg of chicken type II collagen (Chondrex, Redmond, WA, USA) emulsified in complete Freund’s adjuvant and injected into the tail base. The reaction was then boosted with another injection of 50 μg of the collagen in incomplete Freund’s adjuvant on day 21 [11]. Varying by the individual mouse, arthritis developed between day 40 and day 55 in 70% to 80% of the immunized animals. Disease development was defined by footpad swelling and histology. Footpad thickness was measured with a pressure tension caliper in a double-blinded manner to follow the course of inflammation. Arthritis severity was defined by scoring the increase in thickness of the hind footpads, calculated as (thickness at day of measurement minus thickness prior to immunization at day 0) divided by (thickness prior to immunization at day 0). A mouse was considered as arthritic when one of its four footpads had swollen and a hind footpad thickness had increased by 15%. At the end of the experiment, the limbs were harvested for X-ray analysis using a Faxitron for small animals (model 43855; Faxitron Bioptics, Tucson, AZ, USA) and exposed to 35 V for 12 seconds. In addition, the footpads were fixed in 10% neutral buffered formalin, decalcified in 10% ethylenediaminetetraacetic acid, embedded in paraffin, cut into serial sections, stained with hematoxylin and eosin (H&E) and evaluated for tissue destruction, cell infiltration and overall morphology as described by Chan and Moore [17].

### Preparation of PSVue 794 and control dye

The PSVue 794 reagent kit was obtained from Molecular Targeting Technologies, Inc. (West Chester, PA, USA). The dye was prepared according to the manufacturer’s instructions. Briefly, the apo-PSVue 794 powder was dissolved in the supplied diluent to a final concentration of 2 mM solution, which was mixed with an equal volume of 4.2 mM zinc nitrate solution and then placed in a water bath at 37°C to 40°C before use. The control dye, also from Molecular Targeting Technologies, contains the exact same fluorophore but lacks the Zn-DPA targeting moiety. The compound exhibits fluorescence at the NIR region, with an excitation maximum of 787 nm and an emission maximum of 808 nm. It was prepared for injection in the same manner as PSVue 794 [32].

### Preparation of mice for imaging

Mice that had developed arthritis were put on a low-fluorescence AIN-76 diet for 4 to 6 days, and the fur on their legs was removed with Nair (Church & Dwight, Ewing, NJ, USA) approximately 16 hours prior to *in vivo* imaging analysis. Unbound dye is cleared through urination, and the dye may be tracked on the fur. Freshly prepared 1 mM experimental or control dye was administered, in excess, by retro-orbital injection at a volume at 30 μl (about 2.2 μg/g body weight) per mouse [35]. A clearance study was performed by scanning the mice injected with the dye prior to injection and 20 minutes, 24 hours and 48 hours after injection. The unbound dye was allowed 48 hours to clear out of the circulation. Additional imaging was performed after 24 hours to ensure that the unbound dye had been completely cleared during the scan.

A competition between PSVue 794 and annexin V (BD Biosciences, San Jose, CA, USA) was also investigated by NIR imaging. To determine whether the PSVue dye was detecting apoptotic cells in the arthritic joints, a displacement study was performed *in vivo* with annexin V. Immunized mice first received an injection with PSVue 794 dye as described above, and the unbound dye was allowed to clear. On the third day after dye injection, the mice were first scanned to ensure retention of the fluorescent signal, and then 63 μg of annexin V was injected retro-orbitally. After the annexin V injection, the mice were scanned at hourly intervals to determine whether there was displacement of the bound PSVue 794 dye. With another set of immunized mice, the two reagents were given in a reversed order. Annexin V protein was administered retro-orbitally into mice 30 minutes prior to PSVue dye. The mice were then scanned on the next day.

### Imaging

PSVue 794 binding was measured using the LI-COR Odyssey Image System for NIR emission, as instructed in the application protocol manual [36]. Mice were anesthetized by intraperitoneal (IP) injection of a mixture of ketamine (100 mg/kg) and xylazine (5 mg/kg) and placed in the accessory mouse port to keep them at 37°C for scanning. The instrument illuminated at two wavelengths, 700 and 800 nm, and emission fluorescence at 800 nm was collected for analysis. Rectangular areas that covered from the nose to the base of the tail were boxed for scanning. Images were acquired either in 3.5 minutes at 169-μm resolution or in 7 minutes at 337 μm resolution to balance image quality, scan speed and anesthetic limits (less than 10 minutes).

A shaved area close to the region of interest (ROI) was used to set the degree of background autofluorescence for the normalization of each mouse and for the semiquantitative comparison of different experimental subjects. The intensities (counts) registered at the 800-nm wavelength were mapped to a color palette for pseudo-color visual display using the Odyssey Application Software version 3.0. The maximum intensity was set at 4,012 and the gamma value was set at 1 for distributing the color in the pseudo-color image.

## Results

### Correspondence between dye binding and arthritic limbs

**Figure 2:**
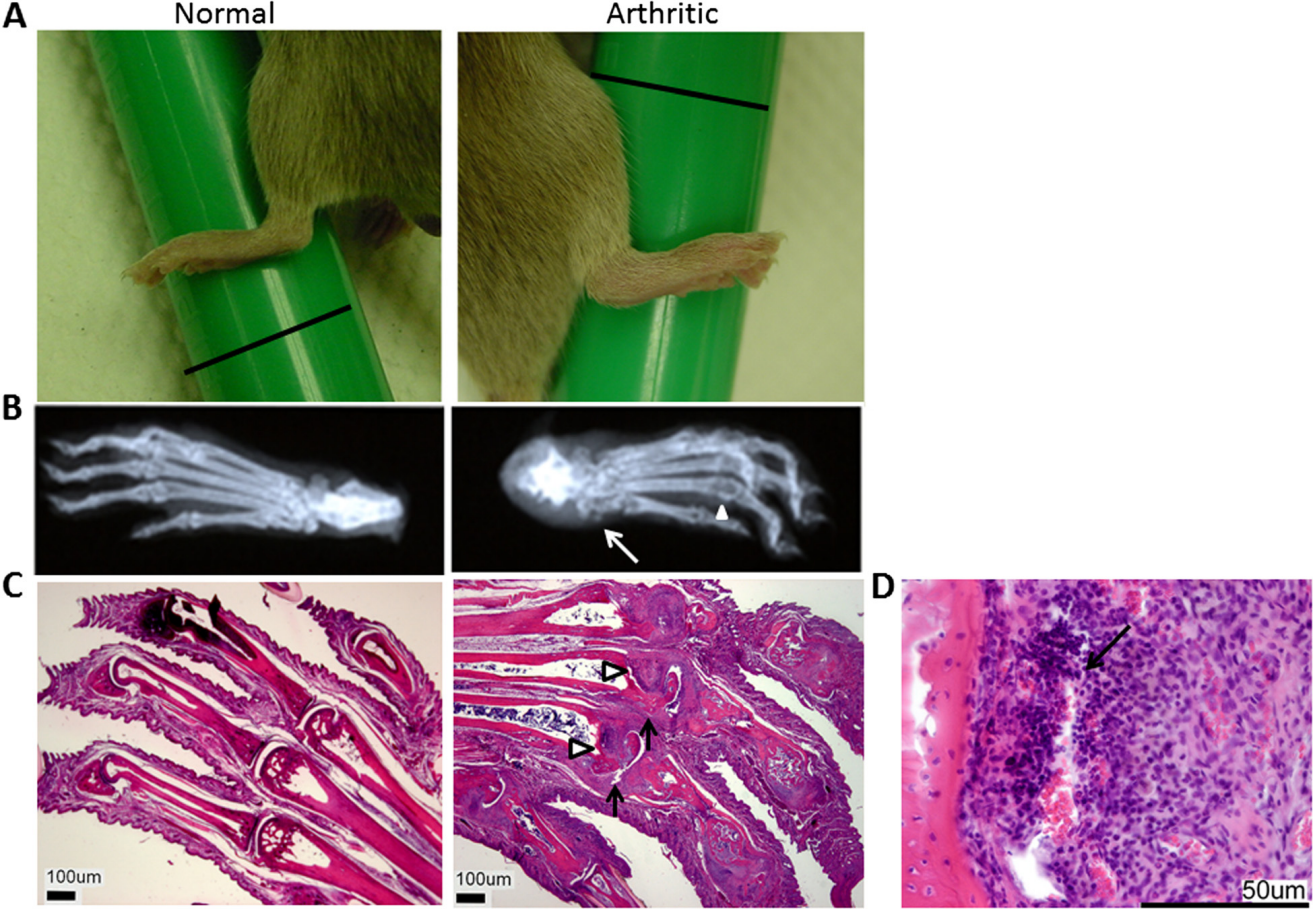
**Morphological characterization of arthritic footpad.** Shown are comparisons of the typical morphology of normal (n = 14) and arthritic (n = 34) footpads. **(A)** Photographs of typical normal and swollen arthritic footpads. The green rods serve as a reference for standardizing the magnification. The black lines on the green rods are 2-cm scale bars. **(B)** X-rays of a normal and a representative arthritic foot. In the latter, the image at right shows soft tissue swelling (arrow) and bone destruction at the ankle as well as loss of bone density and misalignment at the metatarsophalangeal joints (arrowhead). **(C)** Hematoxylin and eosin staining of normal and arthritic animal thin sections. In the latter, the image at right shows bone damage (arrows), cellular infiltrates and soft tissue swelling (arrowheads). Original magnification, ×40. **(D)** A necrotic focus (arrow) adjacent to a bony area within the arthritic joint. Original magnification, ×400.

The pathogenesis of CIA follows three phases: induction, progression and resolution [17]. There is a latency period when the induced mice are asymptomatic, then the thickness of the footpads progressively increases. The swelling reaches a maximum and then begins to subside. In a particular immunization, the group of mice, though similar, has individual variations in latency period as well as in the degree of severity. The manifestation in the hind limbs can be unilateral or bilateral. There are also mice whose hind footpads have not swollen by more than 15% and are considered as non-responders. Morphological and histological details of this CIA model have been presented previously [17]. These differences within a group provide a spectrum of severity for evaluating whether PSVue 794 can be a useful molecular imaging probe for arthritis development. In this study, a total of 34 mice were assigned into groups according to whether none, one or both of their hind footpads had swollen by 15%, with a minimum of 3 per group, and analyzed. The development of arthritis was confirmed with radiography, which displayed a loss of bone density and joint misalignment, and H&E histological staining, which additionally showed hyperplasia and necrosis (Figure 2).

**Figure 3:**
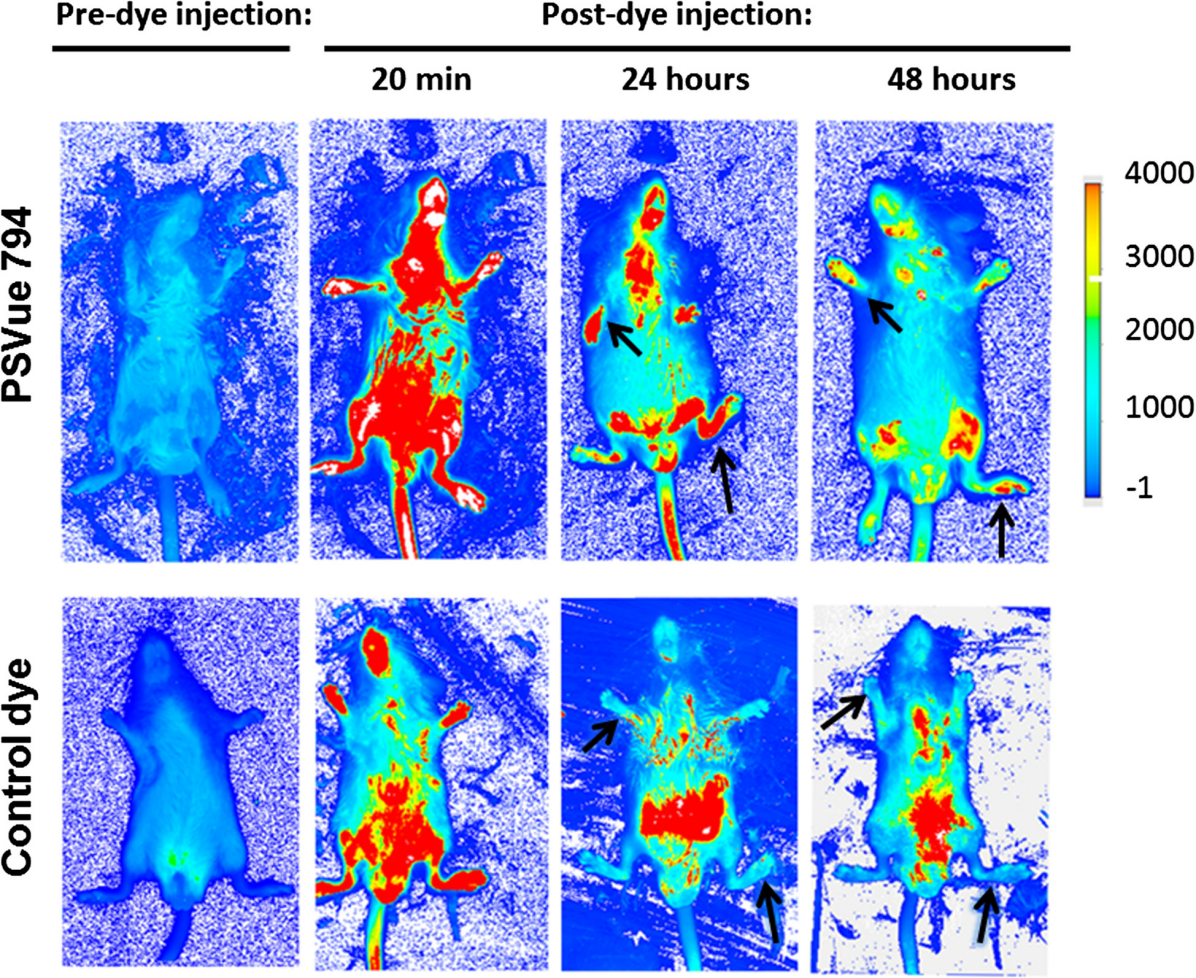
**Clearance study and specificity controls.** Mice were induced for arthritis, and they developed footpad swelling at the level of a 45% to 50% increase in thickness. They were put on an AIN-76A diet for 4 to 6 days and then given 30 μl of 1 mM freshly prepared PSVue 794 (n = 24) or control dye (n = 3) retro-orbitally, as described in the text. The images shown were obtained using the LI-COR Odyssey Infrared Imaging System at the times indicated. The arrows point to the presence or absence of signals in mouse feet using the near-infrared dye and the control dye. The fur at the abdomen of the mice injected with the control dye had not been removed and was contaminated with unbound dye excreted in urine.

Targeting specificity of PSVue 794 for the *in vivo* assay was examined in a clearance study (Figure 3). The arthritic mice, with footpads swollen by 45% to 50% in thickness, were administered either the NIR dye or a control dye that contains the same fluorophore, but without the targeting moiety [32]. Prior to probe injection, emission was at a background level due to autofluorescence. Immediately after intravenous injection of the NIR dye, the level of emission fluorescence was high in the feet and distributed diffusely throughout the whole body. By 24 to 48 hours, excess dye was cleared, and fluorescence was observed only in the legs, where the dye molecule had bound. The legs of the mouse that received the control dye were emitting at background level, indicating that arthritis could be detected only in the presence of Zn-DPA targeting moiety. These results are in agreement with previous reports [32,34].

**Figure 4:**
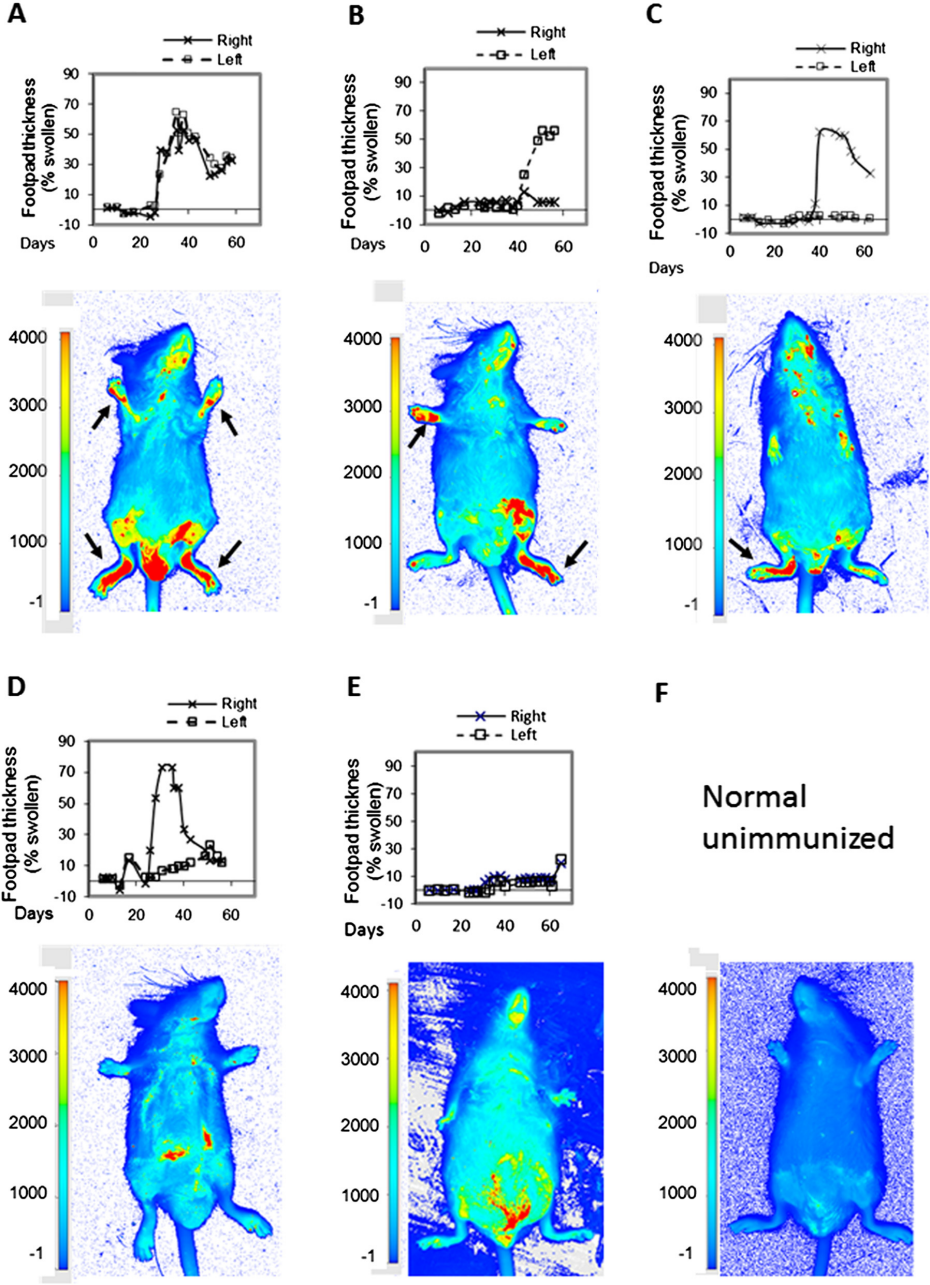
**Correspondence between PSVue 794 near-infrared emission and arthritic footpad swelling.** Mice (n = 34) were induced for arthritis, and hind footpad swelling was followed for 50 to 60 days, as indicated in the line graphs. During the last week of the experiments, the mice were put on an AIN-76A diet for 4 to 6 days and then given 30 μl of 1 mM freshly prepared PSVue 794 retro-orbitally. The images were obtained using the LI-COR Odyssey Infrared Imaging System at 48 hours after PSVue 794 administration. The arrows point to the affected feet. **(A)** A representative mouse with arthritis in both hind feet, swollen by more than 30%, when the image was taken. High fluorescence intensity (red) was detected bilaterally. **(B)** and **(C)** Representative mice with unilateral arthritis in the left or right foot. High fluorescence intensity was detected in the corresponding affected foot. **(D)**, **(E)** and **(F)** show the lack of PSVue 794 near-infrared emission. **(D)** A mouse in which inflammation increased in the right footpad but then subsided. **(E)** A non-responder that did not develop arthritis after immunization. **(F)** A representative unimmunized mouse (n = 10).

The mice were evaluated to determine whether NIR dye binding correlated with arthritis manifestation, as assessed by footpad swelling. The dye provided a semiquantitative marker in correspondence with footpad swelling, as shown in the representative examples in Figure 4. In a mouse whose hind footpads were bilaterally affected, emission was detected in both the left and right limbs. In mice with unilateral manifestation in their hind footpads, emission occurred only in the limbs with arthritis. Emission was not seen in the contralateral limbs, ones with swelling below 10%. In mice with resolved inflammation, as well as the non-responder mice, emission was not detected. It was at the level of background or autofluorescence.

### Displacement and blocking of PSVue 794 by annexin V

**Figure 5:**
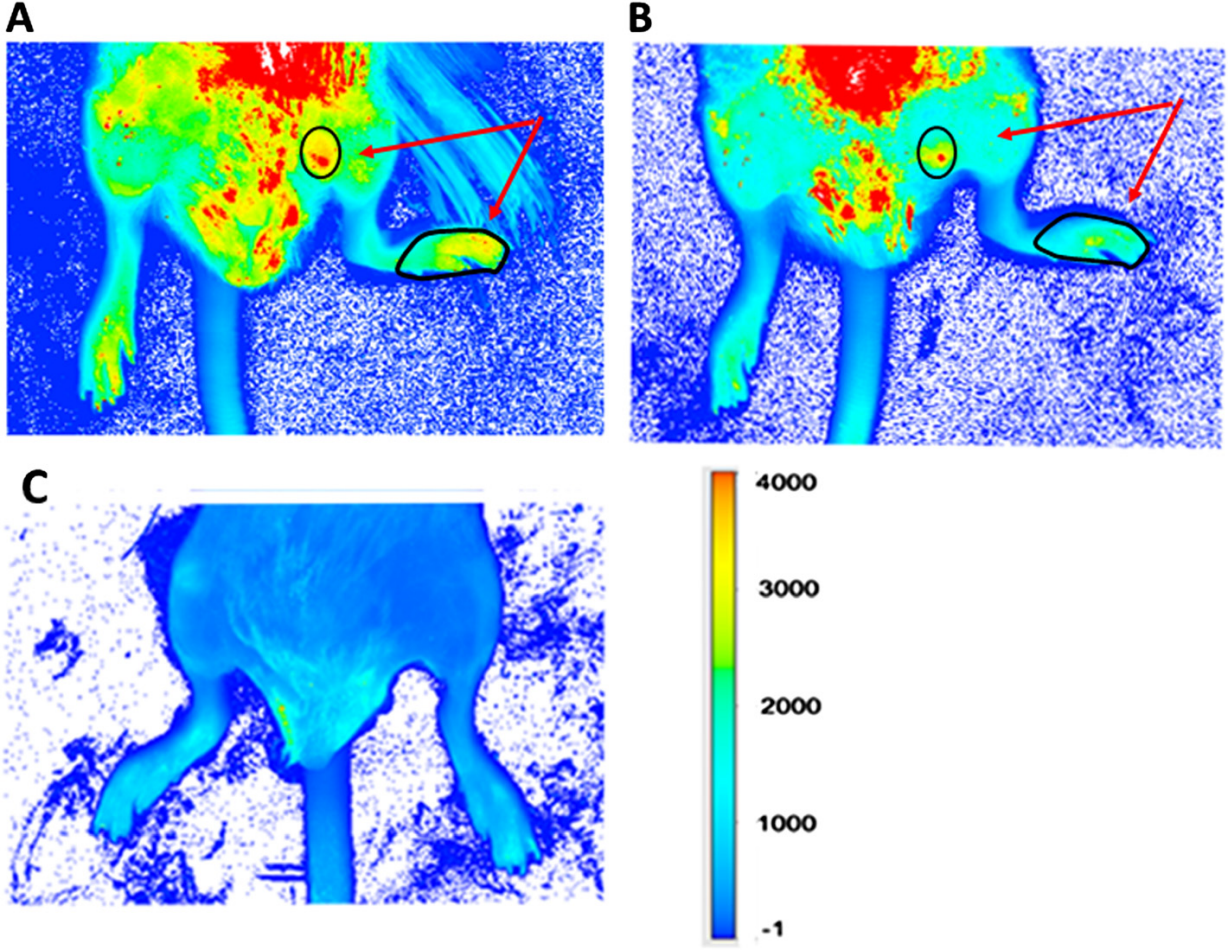
**Displacement and blocking between PSVue 794 and annexin V. (A)** Fluorescence in the hind leg region of a representative mouse (n = 3) whose PSVue 794 dye had been allowed to clear for 3 days before administration of annexin V. **(B)** The signal was reduced at 3 hours after injection of 63 μg of annexin V. After normalization for background, fluorescence emission (K counts and intensity) in the ROIs (circumscribed at the toes and the thigh, indicated by arrows in **(A)** and **(B)**) were compared with the LI-COR Odyssey Infrared Imaging System software, using the average from three scans by analysis of variance. **(C)** When 21 μg of annexin V were administered *in vivo* into a mouse (n = 5) whose footpad was swollen 40%, subsequent injection of PSVue 794, as described in the text, did not result in binding, and the dye was rapidly cleared.

Next, to determine whether the NIR dye was detecting apoptotic cells in the arthritic joints, a displacement study was performed *in vivo* with annexin V (Figure 5). After unbound PSVue 794 had been cleared for 3 days, injection of annexin V led to decease in emission, suggesting that there was a displacement of the PSVue 794 that had been bound to anionic phospholipids. Numerical comparison was performed in two ROIs using the data from three different scans in which the mouse was repositioned. A statistically significant (*P* ≤ 0.05) reduction of NIR emission was observed at 3 hours after annexin V administration. Before injection of annexin V, the ROI at the toes had a K count of 57.6 ± 3.5 and average intensity of 1,385 ± 86. The ROI at the thigh region had a K count of 58.4 ± 7.0 and average intensity of 2,421 ± 252 (Figure 5A). Three hours after injection of annexin V, the K count of the ROI at the toes had decreased to 40.1 + 3.3 and average intensity to 1,172 ± 68. The K count of the ROI at the thigh was reduced to 42.39 ± 2.1 and average intensity to 1,999 ± 83 (Figure 5B). So, annexin V reduced PSVue 794 binding by approximately 30%. When the two reagents were given in a reversed order, emission was non-detectable (Figure 5C). It is likely that PSVue794, as a small molecule, could not bind when the PS on the cellular membrane had become covered by a larger protein. Annexin V complexes with PS via hydrophobic domains [37]. PSVue 794 binds by electrostatic forces to membrane surfaces enriched with PS [29].

**Figure 6:**
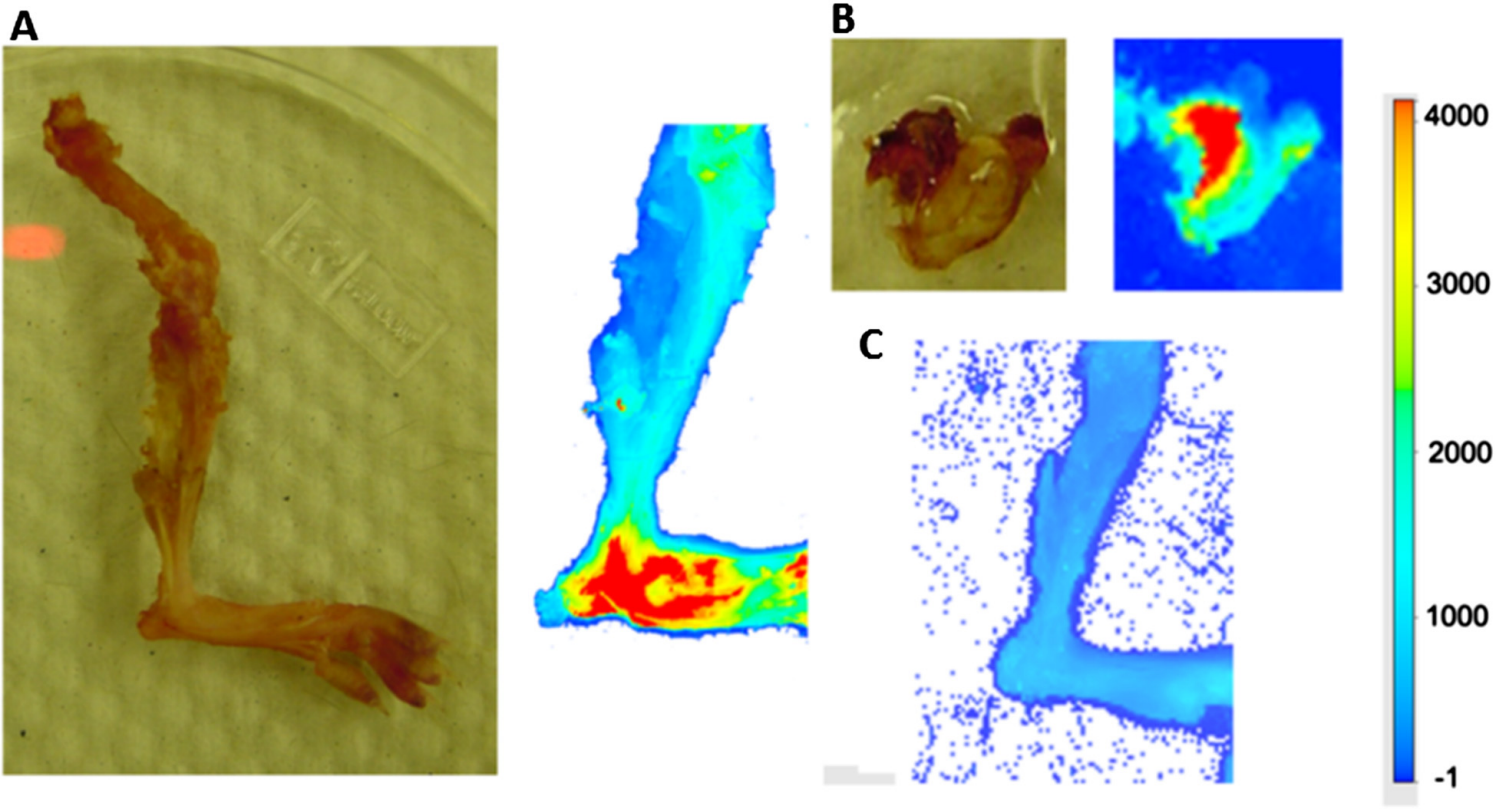
***Ex vivo*** **imaging. (A)** Skin was removed from an amputated arthritic foot in which thickness had increased by 29% (n = 6). High intensity of near-infrared emission was detected in the pseudo-colored image, thus validating that the fluorescence was due to cell death at the swollen ankle rather than to dye tracked externally on the foot. **(B)** Detection of apoptosis in portion of the associated inguinal lymph node. **(C)** Lack of emission in a deskinned arthritic limb from a mouse injected with the control dye (n = 3).

Binding of the NIR dye was also demonstrated *ex vivo*. A swollen foot was amputated, and its skin was removed and then scanned. The emission was retained, ensuring that fluorescence was internal (that is, from the inside). A portion of its draining inguinal lymph node also showed some degree of fluorescence, suggesting the presence of vascular destruction and cell death in the organ. On the contrary, emission was not detected in a similar foot labeled with the control dye (Figure 6).

## Discussion

Arthritis is a complex family of musculoskeletal disorders consisting of more than 100 different diseases or conditions that destroy joints, bones, muscles, cartilage and other connective tissues that hamper or halt physical movement. Among them, RA, triggered by autoimmunity, is one of the most common and the most disabling. This study demonstrates that PSVue 794, by binding to exposed anionic phospholipid PS, provides a cost-effective way to measure inflammation by apoptosis in a murine arthritis model.

Apoptosis is a regulated function in the pathogenesis of arthritis, and annexin V is well recognized as specific for PS and applicable in imaging of apoptosis [38]. Apoptosis occurs when inflammation begins to resolve. If this resolution is obstructed, cell death perpetuates and chronic inflammatory diseases develop. In humans, ^99m^Tc-annexin can be used to visualize arthritic joints and detect onset of bone destruction. Post *et al*. [8] used ^99m^Tc-annexin V and autoradiography to study the extent and severity of apoptosis in the front and rear paws of DBA/1 mice with CIA. Our present study reveals that PSVue 794 mimics the apoptosis-sensing function of annexin V. The dye detects inflammation-associated cell death and works as a probe for non-invasive measurement of arthritis. We also show that the level of emission reflects the degree of disease manifestation in parallel with footpad swelling, a traditional anatomical parameter that requires double-blind measurement because it is more prone to human subjectivity. The difference in fluorescence emission between mice can be compared objectively and semiquantitatively based on a scale of emission. Control studies show that the levels of emission in arthritic mice given the dye without the PS targeting moiety, or in non-arthritic mice given the dye, are substantially lower than in the experimental animals. Furthermore, the present study, for the first time to the best of our knowledge, validates the selectivity of binding of PSVue 794 directly by demonstrating that it interferes with annexin V binding and *vice versa*. Previous *in vitro* and *ex vivo* studies have shown only that the dye and annexin binding co-localize [29,30].

Tissue penetration is a hindering factor in fluorescence imaging. This problem is further complicated by the fact that the vasculature is often compromised by vasculitis in inflammatory autoimmune diseases. PSVue 794 has a low molecular weight, so it can distribute through the circulation with less hindrance than the larger annexin V. Although a direct comparison between the two molecules has yet to be demonstrated in the current arthritis model, the NIR dye has been shown to label human epidermoid carcinoma xenografts with broken blood vessels more efficiently than annexin V [27,32]. Thus, it is likely that a smaller dye molecule would have an advantage over annexin V for imaging inflammatory diseases. A factor underlying this success is that the dye emits in the NIR region, where tissue absorption and scattering are less than visible light. This non-invasive *in vivo* imaging provides a means by which to follow the course of inflammation in real time and may allow investigators to study animals longitudinally without the need to kill them, thus reducing the number of animals required for sequential time point analyses.

The present study was done using an economical imaging system widely used and present in many laboratories—the Odyssey Infrared Imaging System—because arthritis is manifested in the footpads. Thus, the method is affordable for most investigators. In addition to the 800-nm fluorescence intensity utilized in this study, the instrument detects fluorescent dye that emits at 700 nm, thus allowing investigators a range of choices, such as prelabeled markers and dual labeling. The mice were scanned with the mouse port accessory (MousePOD; LI-COR Biosciences), and the emitted fluorescent photons in affected limbs of small animals were counted. Other imaging systems, such as Kodak Image Station 4000 (Eastman Kodak, Rochester, NY, USA), which uses white light and filters, have also been used in conjunction with PSVue 794 [39].

In this study, we performed the scanning without the use of an inhalation anesthesia vaporizer system, further reducing the cost. The scanning was done at 169 μm to accommodate the anesthetic time limits (less than 10 minutes) attained by IP injection of ketamine and xylazine. An anesthetic vaporizer system can be added to lengthen the time for which the mouse can remain motionless, then the scanning can be done at shorter intervals. For those investigators who specialize in *in vivo* imaging of small animals, instruments such as the LI-COR Pearl Impulse detector may be used. This equipment’s scan speed can be maximized to seconds, allowing analysis of large number of mice in a short period of time. In addition, imaging can be done in other models of inflammation where the inflicted organ is internal, such as when apoptosis occurs in internal organs (for example, the brain) [31].

Extending from animal models, the PSVue molecule may be further developed for diagnosis in humans, as annexin V–based apoptosis imaging has produced encouraging results for assessment of chemo- or radiotherapy. Radiolabeling the Zn-DPA targeting moiety with ^18^F and ^64^Cu for positron emission tomography and ^99m^Tc and ^111^In for single-photon emission computed tomography may circumvent the challenges caused by fluorescent signals being attenuated *in vivo*.

## Conclusions

The PS-binding PSVue 794 dye was shown to be capable of measuring arthritis in a rodent model (murine CIA). This fast, sensitive and cost-effective technique may be adaptable for clinical application in patients with RA.

## Abbreviations

CIA: Collagen-induced arthritis
H&E: Hematoxylin and eosin
IP: Intraperitoneal
NIR: Near-infrared
RA: Rheumatoid arthritis
ROI: Region of interest
PS: Phosphatidylserine
Zn-DPA: Bis(zinc^2+^-dipicolylamine)
